# Advancements in Obstructive Sleep Apnea Diagnosis and Screening Through Artificial Intelligence: A Systematic Review

**DOI:** 10.3390/healthcare13020181

**Published:** 2025-01-17

**Authors:** Lucrezia Giorgi, Domiziana Nardelli, Antonio Moffa, Francesco Iafrati, Simone Di Giovanni, Ewa Olszewska, Peter Baptista, Lorenzo Sabatino, Manuele Casale

**Affiliations:** 1 Integrated Therapies in Otolaryngology, Fondazione Policlinico Universitario Campus Bio-Medico, 00128 Rome, Italy; l.giorgi@unicampus.it (L.G.); francesco.iafrati@unicampus.it (F.I.); simone.digiovanni@unicampus.it (S.D.G.); l.sabatino@policlinicocampus.it (L.S.); m.casale@policlinicocampus.it (M.C.); 2 School of Medicine, Università Campus Bio-Medico di Roma, 00128 Rome, Italy; domiziana.nardelli@alcampus.it; 3 Department of Otolaryngology, Sleep Apnea Surgery Center, Medical University of Bialystok, 15-276 Bialystok, Poland; ewa.olszewska@umb.edu.pl; 4 ENT Department, Al Zahra Private Hospital Dubai, Dubai 23614, United Arab Emirates; peterbaptista@gmail.com

**Keywords:** OSA, screening, diagnosis, artificial intelligence

## Abstract

Background: Obstructive sleep apnea (OSA) is a prevalent yet underdiagnosed condition associated with a major healthcare burden. Current diagnostic tools, such as full-night polysomnography (PSG), pose a limited accessibility to diagnosis due to their elevated costs. Recent advances in Artificial Intelligence (AI), including Machine Learning (ML) and deep learning (DL) algorithms, offer novel potential tools for an accurate OSA screening and diagnosis. This systematic review evaluates articles employing AI-powered models for OSA screening and diagnosis in the last decade. Methods: A comprehensive electronic search was performed on PubMed/MEDLINE, Google Scholar, and SCOPUS databases. The included studies were original articles written in English, reporting the use of ML algorithms to diagnose and predict OSA in suspected patients. The last search was performed in June 2024. This systematic review is registered in PROSPERO (Registration ID: CRD42024563059). Results: Sixty-five articles, involving data from 109,046 patients, met the inclusion criteria. Due to the heterogeneity of the algorithms, outcomes were analyzed into six sections (anthropometric indexes, imaging, electrocardiographic signals, respiratory signals, and oximetry and miscellaneous signals). AI algorithms demonstrated significant improvements in OSA detection, with accuracy, sensitivity, and specificity often exceeding traditional tools. In particular, anthropometric indexes were most widely used, especially in logistic regression-powered algorithms. Conclusions: The application of AI algorithms to OSA diagnosis and screening has great potential to improve patient outcomes, increase early detection, and lessen the load on healthcare systems. However, rigorous validation and standardization efforts must be made to standardize datasets.

## 1. Introduction

### 1.1. Obstructive Sleep Apnea: Prevalence and Healthcare Impact

Obstructive Sleep Apnea (OSA) is a common, yet underdiagnosed sleep-related breathing disorder that carries a risk of complications, increases mortality, and causes additional healthcare load. It is characterized by intermittent upper airway obstruction causing interruptions of breath (apneas) and reductions in breath amplitude (hypopneas) during sleep, lasting between 10 s and 60 s or longer [1]. These repetitive episodes of apnea and hypopnea can lead to blood hypoxemia, hypercapnia, fragmented sleep, recurrent nocturnal arousals, enhanced respiratory effort, increased sympathetic nerve activity, and even sudden cardiac death. Indeed, it was shown that OSA has a substantial impact on the cardiovascular system, and it is associated with an increased risk of hypertension, stroke, ischemic heart disease, and venous thromboembolism [2,3]. It affects 24% of men and 9% of women between 30 and 60 years old [4]. According to a recent epidemiological study in Italy, there are 12,329,614 patients affected by moderate to severe OSA (27% of the population) and an overall prevalence of more than 24 million people aged from 15 to 74 years old (54% of the population). However, only 460,000 patients with moderate to severe OSA are diagnosed (4% of the estimated prevalence) and 230,000 are treated (2% of the estimated prevalence), suggesting a substantial gap in the diagnostic and treatment workflow [5].

### 1.2. Current Diagnostic Methods and Challenges

The current gold standard for OSA diagnosis is full-night polysomnography (PSG), which requires the following measurements: electroencephalogram (EEG), electrooculogram (EOG), electrocardiogram (ECG) or heart rate, chin electromyography (EMG), airflow, arterial oxygen saturation, and respiratory effort [6]. However, the full PSG is highly costly due to the numerous measurements required, the need for specialized staff, and the full-time night occupation of the laboratory. Alternatively, unattended tests, called Home Sleep Apnea Tests (HSATs), were recently proposed [7]. Many insurance companies agree to the use of HSATs for OSA diagnosis since the Centers for Medicare and Medicaid Services (CMS) declared in 2008 that the use of home testing is reimbursable [8,9]. These tests do not require sleep laboratories, are easier to perform, less expensive, and are widely available. This novel testing strategy has demonstrated cost-effectiveness from healthcare, societal, and patient perspectives. An Italian study showed that home-based OSA testing reduced direct medical costs by 44%, personal expenses such as productivity loss and travel by 37%, and societal costs by 20% [10]. On top of that, a telemedicine approach represents a patient-friendly solution to those living far away from referral centers, filling the accessibility gap for patients belonging to rural communities [11]. Despite the introduction of HSAT notably shortening the waiting list, this disorder remains highly underdiagnosed and undertreated. Therefore, new solutions are needed to increase the sample of the general population to be diagnosed and treated. In light of these challenges, a clinical prediction model that can accurately identify patients who are most likely to benefit from PSG has to be developed. Such a model should aim to exclude a diagnosis of OSA when the probability is low, establish the likelihood before considering PSG, and prioritize patients requiring PSG based on the probability of a positive outcome. Also, the American Academy of Sleep Medicine (AASM) has called for more robust diagnostic methods that move beyond the Apnea–Hypopnea Index (AHI) alone, incorporating demographic, anthropometric, comorbidity, and symptom data for better screening accuracy [12].

### 1.3. Artificial Intelligence in OSA Screening and Diagnosis

The healthcare industry has witnessed a rapid advancement of Artificial Intelligence (AI) models, which apply statistics and algorithms to analyze complex databases, offering benefits in diagnostics, therapy, and disease prediction in the medical field [13,14]. Among the possible applications, AI can be used to automatically analyze medical images to detect anomalies such as tumors, fractures, or infections with high precision or to predict patient outcomes, such as disease progression or response to treatment, by analyzing historical data and identifying patterns [15]. Also, AI is able to process and analyze unstructured medical records, integrating them with literature research and case reports, enabling the extraction of relevant information and improving the diagnosis process and treatment choice [16,17]. Indeed, AI can further enhance telemedicine by providing initial diagnostic support and automated therapy recommendations, which are then reviewed by physicians. This would accelerate remote interactions between doctors and patients even further. In the otolaryngology field, Machine Learning (ML)- and deep learning (DL)-based predictive models were implemented to refine OSA screening strategies, aiming to enhance sensitivity and accuracy. These models are particularly effective in diagnosing and screening due to their ability to process vast amounts of data and identify patterns that may not be apparent to clinicians. Among the possible models, one of the most used algorithms is Support Vector Machine (SVM), which is based on the concept of margin: this classifier defines a linear decision boundary, and for each data point, the distance from this boundary is calculated, and this distance is referred to as the margin. A larger margin indicates greater confidence in the classification decision. This algorithm offers the significant advantage of being extremely fast during testing due to its low computational demand, enabling deployment across various devices. Another commonly used binary classifier is the logistic regression (LR), a model that predicts the probability that a given observation belongs to a particular class, for example, predicting disease risk. The k-nearest neighbor (kNN) algorithm is a versatile classifier capable of handling both binary and multiclass classification tasks. In binary classification, kNN predicts the class of a new data point based on the classes of its nearest neighbors in the feature space, calculating the distances between the new data point and existing data points in the dataset. For multiclass classification, kNN applies the same principle, considering the classes of the *k* nearest neighbors and assigning the most frequently occurring class. However, kNN is best suited for smaller datasets, where distance calculations are computationally feasible, and the complexity of the decision boundary is a key consideration.

Differently, random forest (RF) is an ensemble learning method that constructs multiple decision trees (DTs) during training and outputs the mode of the classes (classification) or the average prediction (regression) of the individual trees. DTs are just like real trees, with roots, branches, and nodes. The nodes can either be intermediate nodes, which can be seen as roots for subtrees, or terminal nodes, also known as leaves. A DT is essentially a step-by-step questioning process about the objects being analyzed, where each leaf corresponds to a possible class. At each node, a question is asked about a different feature until reaching a leaf node, which represents the final answer. This is suitable for larger datasets, noisy data, and when interpretability of feature importance is desired. Similarly, Extra Trees construct *n* trees from the entire training set, with each tree featuring randomly selected attributes in internal nodes. This inherent randomness facilitates diverse tree classifications within the same dataset, contributing to enhanced ensemble performance. DL is a subset of ML that uses neural networks. These networks are composed of interconnected nodes, called neurons, organized into layers that can automatically learn features from raw data. Neural networks are computational models inspired by how biological neural networks in the human brain process information. Among the DL models, the Convolutional Neural Network (CNN) is a specialized neural network that processes structured grid-like data, such as images and videos. In the literature, several studies were found that apply these types of models to a great variety of data, such as anthropometric data, medical images, and respiratory sounds. For instance, ML algorithms were used to analyze anthropometric measurements to predict the likelihood of OSA, while DL models like CNNs were extensively applied to interpret medical imaging, including radiographs and CT scans, for detecting abnormalities and disease markers. Furthermore, the analysis of respiratory sounds using ML and DL techniques has shown promising results in diagnosing respiratory conditions, demonstrating the versatility and potential of these algorithms in enhancing early detection and accurate diagnosis across various medical conditions. These AI-driven diagnostic tools not only improve the efficiency and accuracy of disease detection but also facilitate personalized treatment plans by providing insights into patient-specific risk factors and disease progression. As a result, AI models are becoming indispensable in modern healthcare, driving forward the capabilities of precision medicine and improving overall patient outcomes. This work aims to systematically review all the original articles that have employed ML and DL algorithms to diagnose and screen patients affected by OSA in the last ten years. The study is divided based on the input data considered into six sections: (1) anthropometric indexes, (2) imaging, (3) electrocardiogram (ECG), (4) respiratory sound, (5) oximetry, and (6) other signals.

## 2. Materials and Methods

### 2.1. General Study Design

The review was conducted in accordance with the guidelines of the Center for Review and Dissemination’s Guidance for Undertaking Review in Health Care. Reporting was aligned with the Preferred Reporting Items for Systematic Review and Meta-Analyses (PRISMA) statement [18]. This study is registered in PROSPERO (Registration ID: CRD42024563059).

### 2.2. Data Sources and Search Strategy

A comprehensive electronic search was carried out on PubMed/MEDLINE, Google Scholar, and SCOPUS databases. An example of a search strategy is the one used for PubMed/MEDLINE: “Artificial intelligence” and “OSA screening”; “Machine learning” and “OSA screening”; “Artificial intelligence” and “OSA diagnosis”; “Machine learning” and “OSA diagnosis”; “Automatic prediction” and “OSA”; “Artificial intelligence” and “OSA classification”. The searching strategies were designed to meet the specific criteria of each database, and cross-referencing was performed to minimize the risk of missing relevant data. The final search was run in June 2024.

### 2.3. Inclusion/Exclusion Criteria

Eligibility criteria were met for articles that presented original data on the use of ML algorithms for diagnosis and predicting OSA in suspected patients. Exclusion criteria included the following: (1) publications in languages other than English; (2) case reports, reviews, conference abstracts, and letters; (3) studies with unclear and/or incomplete data; (4) studies which failed to report the AI algorithm applied to diagnose or screen patients with OSA; (5) articles published before 2014.

### 2.4. Data Extraction and Data Analysis

Initially, all the articles were screened by title and abstract. Full-text versions of each publication were subsequently evaluated, and the studies deemed irrelevant to the subject of this review were excluded. Data extraction from the included articles was carried out systematically using a structured form. A qualitative synthesis analysis was performed on selected studies, focusing on the employment and performance of AI models for the prediction and diagnosis of OSA.

The performance evaluation metrics extracted from the studies included in this review were as follows: area under the curve (AUC), which measures the ability of a model to distinguish between classes, with values closer to 1 indicating better discrimination; accuracy, as the proportion of correctly classified samples; sensitivity, which represents the ability of a model to correctly identify positive cases; specificity, which indicates the ability of a model to correctly identify negative cases; and Interclass Correlation Coefficient (ICC), assessing the reliability or agreement between predicted and actual values, with higher values indicating better reliability.

### 2.5. Statistical Analysis and Summary of Findings

Due to variations in reporting styles and the lack of consistent data across the included studies, a statistical or quantitative analysis of the findings could not be performed. As a result, the effects on the individual outcomes and the overall quality assessments were described narratively. The authors of the included studies were not contacted for additional information.

## 3. Results

**Figure 1:**
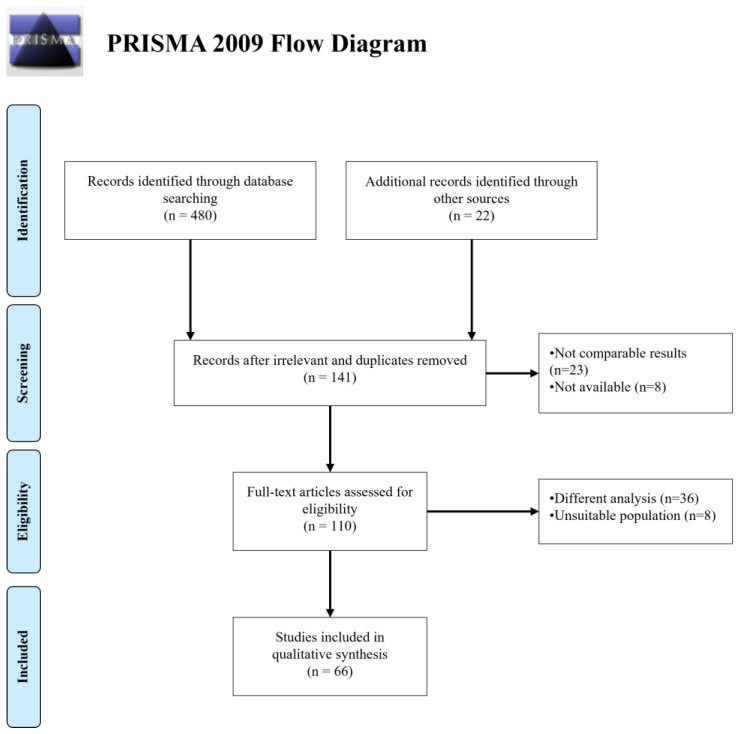
A flowchart outlining the paper selection process of the systematic review (based on PRISMA guidelines).

**Figure 2:**
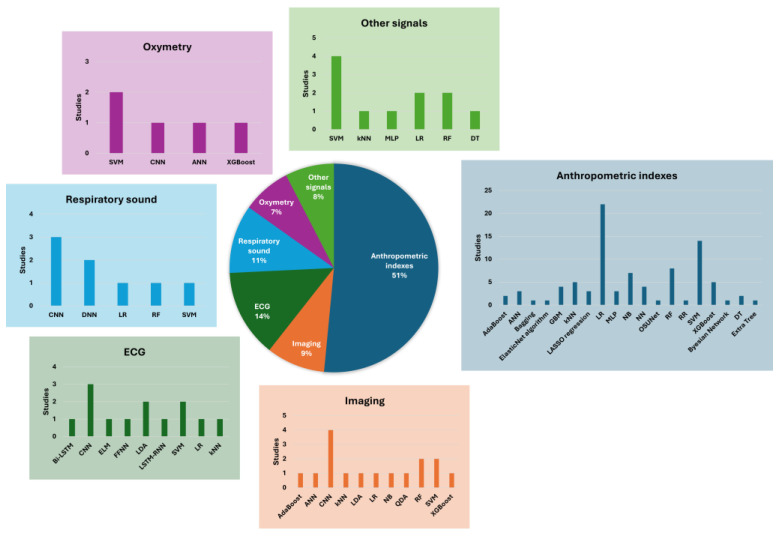
Type of data and algorithms used by included studies.

The search criteria returned 502 articles, among which 361 papers were removed as they were considered irrelevant or duplicates. As further screening occurred, 31 more papers were excluded, resulting in 66 articles that fulfilled the inclusion criteria. A flow diagram of the selection process is shown in Figure 1 (PRISMA flow diagram). The data of 131,823 patients were analyzed in all the studies included. We observed an overlapping of data across the different databases therefore they were considered singularly. In particular, 10,577 patients’ data were taken from the Wisconsin Sleep Cohort (WSC) database [19,20], 10,862 from the Sleep Heart Health Study Visit 1 (SHHS1) [19,21], 621 from SHHS2 [21], 1154 from the Taipei Medical University Hospital (TMUH, Taipei, Taiwan) [22], 5245 from the Shuang-Ho Hospital (SHH, New Taipei City, Taiwan) [22], 369 from Río Hortega University Hospital of Valladolid (UHV) [21], 1463 from the Cleveland Family Study (CFS) [21], 3937 from Osteoporotic Fractures in Men Study (MROS) [21], 2056 from the Multi-Ethnic Study of Atherosclerosis (MESA) [21], 32 from the MIT PhysioNet Apnea–ECG database [23,24,25,26,27,28], and 25 from the University College Dublin Sleep Apnea Database (UCDDB) [27,29]. Figure 2 shows the number of studies for each type of data and the algorithms used by the authors. Detailed tables reporting the characteristics and the outcomes of the included studies can be found in the Supplementary Materials (Tables S1 and S2).

### 3.1. Anthropometric Indexes

Several authors used the anthropometric parameters of the patients, such as age, sex [30], body mass index (BMI) [31], ethnicity [32], and snoring status [33], to build predictive models for OSA. Artificial Neural Network (ANN), LR, and SVM models displayed the AUCs ranging from 0.81 [31] to 0.82 [30], while Kernel SVM (KSVM) displayed an AUC of 0.66 [32]. All models were comparable in terms of efficacy as OSA prediction tools and according to the STOP-BANG questionnaire [30,31,32], which is considered the gold standard screening questionnaire. Application of classifiers, such as Multilayer Perceptron Networks (MLPs), showed an accuracy of 86% in classifying a patient as healthy or being affected by OSA [33]. Likewise, age and sex were analyzed in relation to average systolic blood pressure using a Microsoft decision tree algorithm developed in Microsoft SQL server 2008 business intelligence, obtaining an AUC of 0.99 and an overall accuracy of 96.9% [34]. Moreover, other authors [20,35,36,37,38,39,40,41,42,43,44] implemented their models with further anthropometric variables, such as waist and neck circumference, lifestyle habits and comorbidities, OSA-related symptoms, and sleep questionnaires as input to LR, ANN, SVM, DT, RF, k-NN, neural networks (NNs), Ridge regression (RR), Gradient Boosting Machine (GBM), Light Gradient Boosting Machine (LGBM), Extreme Gradient Boosting (XGB), Adaptive boosting (AdaBoost), Bootstrapped aggregating (Bagging), Gradient Boost DT, Least Absolute Shrinkage and Selection Operator (LASSO) regression, Naïve Bayes (NB), tree-augmented Naïve Bayes (TAN), CatBoost, and MLP. Among all the algorithms, the best performances were obtained for GBM with a maximum AUC of 0.892 in one study [44] and 0.857 [35] in another, and for RF with a partial area under the precision–recall curve of 0.862 [36]. Among these studies, the application of an alternative ensemble technique, the Extra Tree algorithm, is worth noting as its Area Under the Receiver Operating Characteristic curve (AUROC) (0.896), accuracy (90%), and specificity (90%) exceed those of the above-mentioned algorithms [44]. Ferreira-Santos et al. discussed a series of NB and TAN models designed for pre-PSG OSA evaluation [40,41]. Among them, both the TAN algorithm using a set of 38 anthropometric features identified from the literature review obtained the best overall performance, with an AUROC of 0.79 and an accuracy of 72.6%. However, this result was comparable to that obtained using a restricted set of six features to an NB model (AUROC 0.79; 70% accuracy), which is also less cumbersome and lengthy to apply in primary care settings. Also, Manoochehri et al. demonstrated the comparability of an LR model over a DT model in terms of accuracy (74% vs. 76%), specificity (78% vs. 80%), and sensitivity (70% vs. 67%) [37]. In that year, the same group also compared the performance of an LR model built using the best anthropometric variables using Akaike’s information criteria to an SVM model designed with the radial basis function kernel. This time, they showed that the implemented SVM model displayed higher accuracy (80% vs. 73%) and specificity (85% vs. 70%) [38]. Interestingly, two articles utilized the LASSO regression analysis to develop a normogram for OSA prediction [40,43]. Xu and colleagues combined both anthropometric and biochemical features (glucose, insulin, and apolipoprotein B levels), displaying an AUC of 0.84 [39], while Hsu and colleagues opted for anthropometric features alone, displaying an AUC of 0.88 for moderate–severe OSA detection [42]. Likewise, Ge et al. implemented their set of variables with a panel of laboratory indexes, such as glycated hemoglobin, hematocrit, total cholesterol, and triglycerides, together with the presence or absence of common carotid plaques [45]. Across the seven algorithms tested, MLP demonstrated the best performance in terms of AUC (0.94). Bozkurt and colleagues tested five state-of-the-art ML models (Bayesian Network; LR; DT; RF; and NN) using a set of 14 non-PSG variables extracted from the patients’ medical histories, symptoms (ESS), and physical examinations [46]. Performance was stratified in three main settings based on OSA severity (normal/present; normal–mild–moderate–severe; mild–moderate–severe). Overall, the RF algorithm yielded the greatest accuracy (60.9%) across all severity classes. Yet, the greatest AUC (0.85–0.91) was obtained by applying the Bayesian Network to all classes, closely followed by RF (0.82–0.84). Tsai et al. [47] implemented their model with specific indicators of body composition, such as whole body, limbs and trunk fat mass, fat-free mass, muscle mass, fat percentage, basal metabolic rate, and body water information (total body/intracellular/extracellular water), together with an analysis of sleep efficiency, sleep architecture, and sleep quality indexes, to predict the risk of moderate–severe and severe OSA. Several machine learning models were utilized, with the RF-based prediction model demonstrating the highest accuracy and AUC (0.90 for moderate–severe OSA and 0.81 for severe OSA). One year later, the same research group [48] implemented the aforementioned features with snoring events obtained through a piezoelectric vibration sensor placed on the triangle of the neck. It emerged that all the above-mentioned features entertain a statistically significant correlation with AHI and ODI (all *p* < 0.01), except for the extracellular to intracellular water ratio (*p* < 0.05). Also, in this study, RF demonstrated the highest accuracy in classifying moderate–severe and severe OSA, being, respectively, 79.32% and 74.37%, respectively. Awakening due to the sound of snoring, witnessing snore, nocturia, restless sleep, and back pain were other informative self-reported factors that demonstrated good efficiency in predicting OSA [49]. SVM, with a 93.4% sensitivity, better predicted the majority of OSA patients, while NB, with 59.5% specificity, predicted healthy people better than the other models. The NB and LR classifiers had the highest AUCs with 0.768 and 0.761, respectively. According to the results, the neural network had a better classification accuracy in the assessed models. Conversely, a set of four questions was added to basic features (age, BMI, neck circumference, history of diabetes mellitus, or hypertension) to develop an ANN-based prediction tool named OsuNet [50]. The four questions concerned witnessed snoring, witnessed apnea, restless leg syndrome, and loss of libido. The OsuNet model displayed a positive likelihood ratio exceeding that of well-known prediction tools, like the STOP-BANG questionnaire (3.4 vs. 1.4). Similarly, age, sex, BMI, comorbidities, and smoking were fed to a Supersparse Linear Integer Model to build a clinical OSA prediction model based on features retrievable in the patient’s clinical history [51]. This model also obtained a positive likelihood ratio exceeding that of the STOP-BANG questionnaire. Machine learning approaches were also applied to identify individuals at severe risk of OSA based on clinical suspicion. Age, sex, BMI, diabetes, anxiety/depression, choking, and septal deviation were selected as features to be fed to several algorithms, using two thresholds: one to identify moderate–severe OSA and one for severe OSA. The SVM model showed a sensitivity of 93% and a specificity of 80%, while reduced LR demonstrated a sensitivity of 79% and a specificity of 56% [52]. Other authors employed ML to implement questionnaires for OSA screening. One example is the BASH-GN questionnaire, which takes into account age, sex, BMI, neck circumference, hypertension, and snoring loudness into six questions [19], while another one is the Digital Sleep Questionnaire, an ML-powered questionnaire built to identify common sleep disorders (OSA, insomnia, delayed sleep phase, and insufficient sleep syndrome) on the basis of 34 questions concerning sleep quality [53]. Both have outperformed well-known questionnaires (STOP-BANG, Epworth Sleepiness Scale-ESS, Berlin, and Functional Outcomes of Sleep Questionnaire—FOSQ), showing an AUC of 0.77 and 0.85, respectively. Zhang and colleagues [54] developed a model integrating sex, age, BMI, neck circumference, and waist circumference to two faciocervical measurements, being the maximum interincisal distance and ratio of height to thyrosternum distance (SABIHC2 model). This set of data, when powered by a multiview CNN, yielded an AUC of 0.83. Yet, when neck circumference, waist circumference, and BMI were present alone in an SVM-powered model, an AUC of 0.88 in women and an AUC of 0.85 in men were obtained [22]. Faciocervical measurements were also employed by Sutherland et al. to predict OSA in a Chinese population. When using an LR-powered model computing face width, cervicomental angle, and BMI, an AUROC of 0.77 was obtained, while an AUROC of 0.81 was obtained by computing cricomental space area, mandibular width, and mandibular plane angle in a classification and regression tree model [55]. Age, sex, BMI, neck circumference, waist circumference, and question eight in the Snore Outcome Survey questionnaire (“Please describe when you snore”) were integrated into an OSA prediction model powered by SVM, NN, and multiple logistic regression (MLR), displaying an AUC of 0.84, 0.83, and 0.83, respectively [56]. The integration of updated Friedman tongue position and tonsil size grading scored an AUROC of 0.80 in predicting the presence of OSA and an AUROC of 0.82 in predicting moderate–severe OSA in a multiple linear regression model enquired by Lin and colleagues [57], and an AUC of 0.84 in a linear regression model applied by Park et al. [58]. When adding height, body weight, neck circumference, waist circumference, hip circumference, ESS, snoring status, and daytime sleepiness to the above-mentioned indexes, in a LR- and SVM-powered smartphone app, AUCs of 60.8% and 62.2% were achieved, respectively [59]. Other surrogates of well-known OSA biomarkers, such as neck grasp in place of neck circumference, proved not to be valid independent predictors of OSA, with an AUC of 0.62 and a specificity of 39.6% in an LR model [60].

### 3.2. Imaging

Lateral cephalograms are a readily available and inexpensive radiographic tool that reveals the characteristics of upper airway configuration, which confers an informative value in OSA detection. When applied to lateral cephalogram interpretation in OSA screening, deep CNN displayed an AUROC ranging from 0.82 up to 0.99 in severe forms [61,62]. Interestingly, Tsuiki et al. [63] have demonstrated that different cephalometric regions correlated with different performance results, reporting an AUROC of 0.92 for the main region, 0.89 for the full image, 0.70 for the head only, and 0.75 for manual cephalometric analysis. ML models were also applied to diffusor tensor imaging for screening purposes, resulting in an AUROC of 0.85 for RF and 0.84 when using SVM [64]. The combination of 3D geometric morphometrics using different ML algorithms has proved to be a rapid, effective, and inexpensive screening tool in two studies. It demonstrated an AUROC ranging from 0.69 when using a multiview CNN-powered algorithm [65], and then to 0.70 when using LR, SVM, AdaBoost Extra Trees, or Linear Discriminant Analysis [66], which further increased to 0.75 by adding the patient’s anthropometric information (age, BMI, neck circumference, waist circumference, hip circumference, hypertension, Mallampati class, and witnessed apnea and sleepiness while driving), regardless of the algorithm employed [66].

### 3.3. Electrocardiographic Signals

Short-term heart rate variability (HRV) signal is able to reveal physiological changes correlated with apnea events in OSA and is readily extracted from electrocardiograms (ECGs). HRV fluctuations in the public MIT PhysioNet dataset were studied by several authors [23,24,25,26,27,28]. A sequence of one-dimensional HRV signals with their features (time–frequency domains, sample entropy, detrended fluctuation analysis) and a two-dimensional HRV time–frequency spectrum image served as the inputs of one model, powered by parallel hybrid deep learning algorithms, namely Bidirectional Long Short-Term Memory (Bi-LSTM) and the SqueezeNet model, which showed a sensitivity of 95.7% by using Bi-LSTM or SqueezeNet alone, and of 100% when using a combination of the two, which was also achieved in terms of accuracy and sensitivity [26]. Conversely, a CNN model consisting of 10 identical CNN-based feature extraction layers, a flattened layer, four identical classification layers mainly composed of fully connected networks, and a Softmax classification layer was used in a further study, showing a 97.1% per-recording accuracy and an 87.9% per-minute accuracy [23]. The same research group applied this algorithm on 15 sub-band signals, achieving a 100% per-recording accuracy and an 85.5% per-minute accuracy in the mid–high frequency band of 31.25–37.5 Hz [24]. Once more, a fast approximation method for principal component analysis (PCA) applied to ECG-derived respiration for OSA detection was presented by Sadr et al., who employed Extreme Learning Machine (ELM) and Linear Discriminant (LD) as classifiers. Their model showed an accuracy of 76.4% by the ELM classifier and an accuracy of 78.4% by LD [25]. HRV was also extracted from a single-channel piezo-electric sensor and combined with the Snoring Index to identify suspected regions of OSA in an SVM model, displaying accuracy of 71.5%, 80.0%, and 71.9% in mild, moderate, and severe OSA detection, respectively [67]. Lastly, nine ECG-extracted features were fed to six classifiers optimized using hyper-parameter models (DT, discriminate analysis (DA), NB, kNN, ensemble DT, and SVM) to perform an automated OSA diagnosis using the PhysioNet dataset [28]. The highest performances were obtained using optimized classifiers, especially kNN and ensemble DT, which scored an AUC of 68.2% [28]. The UCDDB public dataset was employed by Prucnal et al. [29], who operated a Feedforward Neural Network (FFNN) to analyze statistical features extracted from the EEG epochs by combined discrete wavelet transform (DWT) and Hilbert transform (HT), with an accuracy of 77.3%. Lastly, Hu and colleagues [27] applied a CNN-based auto-encoder with a modified training objective to detect OSA in a single-lead ECG in both the PhysioNet and UCDDB datasets, obtaining an AUC of 0.881. HRV was combined with signals from thoracic triaxial accelerometers and pulse oximetry (SpO_2_) in an LSTM-RNN-based model developed for OSA screening and event detection, which demonstrated an overall accuracy of 92.3% in detecting OSA events and 83.9% in AHI severity classification [68].

### 3.4. Respiratory Sounds

Microphones are widely used to detect breathing sounds, snoring sounds, and breathing pauses and analyze breathing cessation (quiet time) or breath reduction between breaths and/or snores, recovery breath gasp after apnea, and modulated breathing patterns. Two public and one self-recorded datasets were fed to a deep CNN algorithm, which was trained to recognize respiration sounds in sleep sound signals and to detect them using an LR classifier to identify OSA patients from potential patients. Using PSG as a reference, the authors obtained an AUC ranging from 0.79 to 0.82 [69]. Similarly, the deep CNN model built by Le et al. [70] on the basis of PSG audio datasets, smartphone audio datasets synced with PSG, and a home noise dataset to train the model to detect OSA reached an accuracy of 86%. The same model was used to classify OSA severity with a sensitivity and specificity of 85% and 84%. Using the same algorithm, the authors tested the performance of the model with sound recording using an Android phone and an iOS one. They demonstrated comparable sensitivity, specificity, and accuracy for OSA screening, being 93.3%, 94.4%, and 94.3% in severe OSA screening using the iOS phone and 92.9%, 94.3%, and 94.1% using the Android phone [71]. Especially in post-pandemic settings, several apps designed for at-home acoustic OSA screening were developed, with the advantage of requiring no further hardware than a smartphone. Among them, the DNN-powered Firefly and Sleep Study apps showed an AUROC of 0.84 to 0.92 in screening moderate–severe OSA [72,73]. Both of these apps are supported by Android and Apple smartphones. Bahr-Hamm et al. [74] combined the entropy of snoring sound, low-frequency ECG-VLF, and thoraco-abdominal effort–PSG signal entropy values as surrogate markers for OSA detection and OSA severity classification using an SVM algorithm. The best performances were obtained using snoring signal entropy and the second night’s data. Lastly, Hajipour et al. [75] compared the performance of RF against LR as feature selection tools and classification approaches for wakefulness OSA screening using daytime tracheal breathing sounds. RF outperformed LR in terms of blind-testing accuracy, specificity, and sensitivity, showing 3.5%, 2.4%, and 3.7% improvements, respectively. However, the regularized LR appeared to be faster than the RF and resulted in a more efficient model.

### 3.5. Oximetry

Oxygen desaturation index (ODI) is historically a robust single oximetry-based feature for OSA screening. One study evaluated a deep learning algorithm, called OxiNet, for AHI estimation from the oximetry signal and evaluated its performance across ethnicity, age, sex, and comorbidities, demonstrating an ICC ranging from 0.92 to 0.94 when tested on several public datasets [21]. Likewise, Hang and colleagues demonstrated an accuracy of 90% and of 87% in an SVM model based on ODI features alone for the diagnosis of severe and moderate–severe OSA, respectively [76]. Conversely, the joint input of dual-channel simultaneous SpO_2_ and airflow recordings were applied to a further SVM model built for at-home OSA screening, with an AUC of 0.98 for moderate–severe AHI detection, outperforming both single-channel SpO_2_ and airflow (AUC of 0.91–0.92) [77]. SpO_2_ was also employed in a different ANN-powered algorithm to estimate AHI and ODI, demonstrating an ability to correctly classify OSA patients on the basis of AHI and ODI, with an accuracy of 90.0% and 94.4%, respectively [78]. Notably, even an XGBoost model combining basic peripheral oxygen saturimetry with simple anthropometric variables (age, sex, height, weight) and respiratory and heart rates yielded an accuracy of 60–77% across both internal and public datasets in a recent study by Talukder and colleagues [79].

### 3.6. Other Signals

Differently from the other included studies, Kang and colleagues [80] developed an algorithm that enables automatic sleep stage classification utilizing frequency–domain features of the sleep EEG, testing three different algorithms: SVM, k-NN, and MLP. MLP yielded the best performance out of the three, with an accuracy of 73% both in sleep stage classification and OSA screening. Differently, Shafiee et al. [81] carried out a feasibility study in three patients, testing a multi-channel ultrasonic OSA detection system based on wavelet-based, temporal, and spectral features extracted from multiple ultrasound waves transmitted through the patient’s neck during sleep. When powered by SVM and Finite State Machine temporary labels, an accuracy ranging from 73.4% to 79.1% was noted across the patients. Lastly, Mosquera-Lopez and colleagues utilized a contactless custom-built device located underneath the patient’s mattress to capture breathing and movement signals (sampling rate of 250 Hz). The system was endowed with DT and LR models to perform an automatic at-home detection of OSA and severity classification, revealing a correct OSA detection rate of 82.9% and a severity classification accuracy of 74.3%. Importantly, most of the participants found the system easy to install and found that the bed felt stable and comfortable while the device was installed [82]. In a further study, data from spirometry (forced expiratory volume/forced vital capacity) and blood gas analyses (partial pressure of oxygen and carbon dioxide) were integrated into anthropometric indexes, comorbidity status, and indicators of snoring and daytime sleepiness to develop a model for OSA severity prediction. When using an eight-feature SVM model, an AUC of 0.65 was observed, which decreased to 0.62 when using only three features. Likewise, RF obtained an AUC of 0.64 [83]. Lastly, one study explored the possibility of using awake negative pressure testing as a tool to screen healthy subjects from OSA patients. Negative pressure was generated using an air amplifier attached to a nasal mask or mouthpiece. Features were extracted from the waveform. The best performance was obtained using RF, reaching an AUC of 0.80 when considering an AHI cut-off of 10 and a nose-negative pressure of −5 cmH_2_O [84].

## 4. Discussion

This systematic review highlights the promising results of AI algorithms in diagnosing and screening OSA. We analyzed 66 original articles published in the last ten years, showing a significant variety in the types of input data used, the algorithms applied, and the resultant diagnostic performance metrics. The use of different ML and DL algorithms for diagnosing and screening sleep apnea varies depending on the type of signal analyzed due to the data’s intrinsic characteristics and the algorithms’ specific capabilities. Among the DL algorithms, CNNs were the most commonly used when employing more computationally onerous data, such as clinical images (4/6), ECG signals (3/9), and respiratory sounds (3/7). Indeed, CNNs can automatically extract relevant features from raw data, reducing the need for manual pre-processing.

### 4.1. Performances and Advantages of Employing ML and DL Algorithms

#### 4.1.1. Anthropometric Indexes

Of the included studies, 31 used anthropometric indexes as features, such as BMI, neck circumference, age, weight, and height. These are numerical data that often exhibit a linear or quasi-linear relationship with the risk of OSA. Also, these types of data are easy to collect, so it was possible to include a large number of patients, ranging from 167 [60] to 17,731 [32]. The most commonly employed algorithm is LR (20/31) since this algorithm is well suited when there is a linear relationship between the independent (anthropometric indexes) and the dependent (risk of apnea) variables. With this type of data, SVM (13/31) algorithms are powerful for classification, especially with limited features. Additionally, SVMs are robust against overfitting, particularly in contexts with high-dimensional data and few samples. Overall, using anthropometric indexes in ML and DL models for OSA screening has shown considerable results. The collection of data such as age, sex, BMI, daily habits such as smoking or alcohol, and comorbidities is common in clinical practice. Because of this, several authors [21,22,30,31,32,33,34,42,43,49,50,51] decided to predict OSA using these variables. The outcomes demonstrated strong performance in terms of sensitivity (68–98%), specificity (66–93%), and accuracy (67–97%). Comparable results were obtained from other studies [36,37,38,39,41,45,47,53,55,56,58,59,60] that have included additional, more OSA-specific tests, like the Friedman tonsils score, the Mallampati score, the endoscopic lingual tonsils score, and symptom questionnaires, to these data (accuracy: 66–89%; sensitivity: 71–94%; specificity: 63–89%). Lastly, some research [20,39,45,47,48] includes body composition measurements and blood analyses, but they did not improve the results of earlier studies (accuracy: 66–90%; sensitivity: 67–92%; specificity: 50–77%).

However, the studies present highly heterogeneous analyses, using combinations of data that vary significantly and often testing different algorithms or combinations of algorithms to achieve the reported results. For this reason, it was not possible to identify the best-performing algorithm, the optimal subset of data, or which factors have the greatest influence on the outcomes.

#### 4.1.2. Imaging

The studies that employed imaging for detecting and screening OSA managed to include a large set of data, ranging from 155 [64] to 5591 [62]. The application of CNNs to imaging data, including lateral cephalograms and 3D geometric morphometrics, revealed high diagnostic accuracy, ranging from 67% to 93% [61,62,63,65]. Also, good results were found using ML algorithms showing an accuracy higher than 73% [64,66]. The results highlight the value of integrating advanced imaging techniques with ML algorithms to enhance diagnostic accuracy. However, the dependency on specialized imaging equipment and expertise may restrict the widespread adoption of these approaches.

#### 4.1.3. Cardiac-Related Parameters

The rationale for using cardiac-related parameters for OSA screening and diagnosis lies in the close correlation between OSA and cardiovascular diseases (CVDs). Repeated apnea and hypopnea episodes result in hypoxemia, hypercapnia, increased respiratory effort, sleep fragmentation, frequent nocturnal awakenings, and, importantly, increased sympathetic activity. Indeed, OSA prevalence ranges from 40 to 80% in subjects affected by heart failure, coronary heart disease, pulmonary hypertension, atrial fibrillation, and stroke [85]. Moreover, OSA prevalence nears 30% in hypertensive subjects and 80% in patients affected by resistant hypertension [86]. Most often, patients are unaware of the long- and short-term cardiovascular correlations of OSA and tend to underestimate the severity of their condition. Therefore, they are largely understudied. Conversely, subjects suffering from CVDs are seldom screened for sleep-related breathing disorders. ECG signals, particularly heart rate variability, were effectively used as input in ML and DL models for OSA detection. In these studies, a smaller amount of data was used, ranging from 25 [29] to a maximum of 115 [23] samples. The best performance was found using CNN and hybrid deep learning algorithms, such as LSTM-RNN, Bi-LSTM, and SqueezeNet, reaching an accuracy higher than 84% [23,24,26,27,68]. Also, good performance was achieved by ML algorithms such as SVM, LR, LDA, KNN, and NB, with an accuracy ranging between 70% and 80% [28,67]. Additionally, six studies employed the public MIT PhysioNet dataset [23,24,25,26,27,28], applying different algorithms to these data. Considering this subgroup of studies, the best performance was achieved by Sheta et al. [28], reaching an accuracy of 91.50%, sensitivity of 91.04%, and specificity of 91.96% in OSA segment detection when combining the Bi-LSTM and SqueezeNet. Also, this combination showed the best OSA recognition accuracy with each record correctly distinguished. The efficacy of these models highlights the potential of features derived from ECGs as easily accessible and noninvasive biomarkers for OSA screening.

Like ECG, oximetry-based features are also widely used for OSA screening. Two studies chose SVM, reaching an accuracy of 90% and 87% based on ODI features alone for the diagnosis of severe and moderate–severe OSA, respectively [76], and of 90.6% to 95.8% when combining SpO_2_ and airflow [77]. The other included studies using deep learning algorithms, OxiNet, and ANN, obtaining, respectively, an ICC ranging from 0.92 to 0.94 when tested on several public datasets [21] and an accuracy of 94.4% [78]. Lastly, an XGBoost model combined oxygen saturimetry features with anthropometric variables and respiratory and heart rates, showing an accuracy of 60–77% [79].

#### 4.1.4. Respiratory Sounds

Among the possible contactless measurements related to this disease, OSA is a condition characterized by distinct acoustic features such as snores, gasps, chokes, and even periods of silence (cessation of breathing). Since these breathing sounds reflect variations in airway patency, they represent valid indicators of respiratory events. Compared to wakefulness, breathing sounds become louder during sleep due to increased collapsibility of the upper airways, resulting from the reduced activity of the upper airway dilator muscles. However, when an apnea event occurs, no breathing sound is audible due to the complete cessation of airflow. Yet, when breathing resumes after an apnea event, the reopening of the airways causes a loud breathing noise. In contrast, hypopneas entail a narrowing of the airway caliber without the characteristic vibratory component of apneas and are therefore identifiable by narrower and irregular breathing sounds [87]. To detect the snoring sound of the patients, most of the authors used audio recordings using a smartphone positioned next to the bed [71,72,73]. Differently, one study [70] used audio from the in-laboratory PSG microphone installed on the ceiling, and part of the recording was taken from the smartphone microphone beside the bed. All these studies analyzed respiratory sounds using DL models and demonstrated high accuracy (> 70%) [69,70,71,72] in OSA detection, especially in the case of severe OSA when the accuracy reached 94% [71]. Lastly, one study [75] used a small Sony ECM-77B microphone embedded in a chamber of 2 mm diameter and placed it over the suprasternal notch of the patient’s trachea to detect daytime tracheal breathing sounds. Interestingly, this study analyzed daytime breathing by asking the participants to breathe five times through their mouth followed by five breaths through their nose with their mouth closed. The participants then underwent an overnight PSG assessment and, based on AHI, were divided into three groups: non-OSA, mild OSA, and moderate/severe OSA. The two algorithms tested, RF and LR, showed, respectively, a specificity of 79.5% and 75.8%, a sensitivity of 84.2% and 82.2%, and an accuracy of 82.1% and 79.3% on the testing set. The results are very promising considering that the tests performed are noninvasive, rapid, and can be carried out in daily clinical routines. However, the variability in ambient noise and recording quality can challenge the robustness and reliability of these models during the daytime and night. Continued refinement of noise reduction and sound processing techniques will be crucial to enhancing the accuracy and reliability of smartphone-based respiratory sound analyses.

### 4.2. Limitations

High-dimensional databases produce sparse data overall, which makes it difficult to identify meaningful patterns and models that might fit noise rather than signal and learn from the peculiarities of training data that do not generalize well. Furthermore, as dimensions grow, processing and analysis become exponentially harder. For example, from a computational point of view, kNN is a very onerous algorithm because it is based on the calculation of the distances between the samples in the feature space and the comparison of all the possible distances to find the most accurate class. Indeed, the computational time increases with the number of samples analyzed. For this reason, this method is not used on datasets with more than 1000–2000 samples. Differently, when implementing a decision tree, during the training phase, it is important to consider the time complexity, which depends on the number of samples, the number of features, the time required for sorting and splitting the nodes, and the space complexity, which is proportional to the number of samples. On the other hand, the computational complexity during the testing phase is significantly lower. Therefore, once the decision tree is trained, it can run efficiently on a mobile device. Likewise, SVMs have the advantage of being extremely fast in the testing phase as they have a computational demand on the order of the number of features. Lastly, the computational complexity of CNNs depends strongly on the depth of the network, the number of input samples, and the parameters of the filters. So, it is important to remember that the burden of the algorithm also affects power consumption, which cannot be neglected if the algorithm is run on a mobile phone, for example, as in the case of automatic recognition applications. This review underscores the diverse and innovative approaches being explored in the application of ML and DL for OSA diagnosis and screening. The strengths of these approaches lie in their ability to handle large datasets, identify complex patterns, and improve diagnostic accuracy beyond traditional methods. However, the heterogeneity in study designs, datasets, and evaluation metrics complicates direct comparisons across studies.

## 5. Conclusions

The application of AI algorithms to OSA diagnosis and screening has great potential to improve patient outcomes, increase early detection, and lessen the load on healthcare systems. To fully utilize these cutting-edge diagnostic tools, this field must continue to progress, and rigorous validation and standardization efforts must be made. A limit of this study is the heterogeneity of the yielded data, as well as the lack of an internationally validated dataset to track a conspicuous number of records to help the development of this interesting research field.

## Data Availability

No new data were created or analyzed in this study.

## Supplementary Materials

The following supporting information can be downloaded at: https://www.mdpi.com/article/10.3390/healthcare13020181/s1. Table S1: The demographic characteristics of the patients included in the studies and of the algorithms used.; Table S2: Outcomes of included studies.

## Abbreviations

AdaBoost	Adaptive boosting
ANN	Artificial Neural Network
AUC	area under the curve
AUROC	Area Under the Receiver Operating Curve
Bagging	Bootstrapped aggregating
Bi-LSTM	Bidirectional Long Short-Term Memory
BMI	body mass index
CC	Correlation Coefficient
CNN	Convolutional Neural Network
DWT	discrete wavelet transform
ELM	Extreme Learning Machine
FFNN	Feedforward Neural Network
GBM	Gradient Boosting Machine
HRV	heart rate variability
HT	Hilbert transform
ICC	Interclass Correlation Coefficient
kNN	k-nearest neighbor
KSVM	Kernel SVM
LASSO	Least Absolute Shrinkage and Selection Operator
LD	Linear Discriminant
LGBM	Light Gradient Boosting Machine
LR	Logistic regression
ML	Machine Learning
MLPs	Multilayer Perceptron Networks
NB	Naïve Bayes
OSA	Obstructive Sleep Apnea
PCA	principal component analysis
QD	Quadratic Discriminant
RF	random forest
RR	Ridge regression
SVM	Support Vector Machine
TAN	tree-augmented Naïve Bayes
XGB	Extreme Gradient Boosting
